# Interface Engineering with Dynamics‐Mechanics Coupling for Highly Reactive and Reversible Aqueous Zinc‐Ion Batteries

**DOI:** 10.1002/advs.202306656

**Published:** 2023-12-02

**Authors:** Qianqian Meng, Xiaoyu Jin, Nuo Chen, Anbin Zhou, Huirong Wang, Ning Zhang, Zhihang Song, Yongxin Huang, Li Li, Feng Wu, Renjie Chen

**Affiliations:** ^1^ Beijing Key Laboratory of Environmental Science and Engineering School of Materials Science & Engineering Beijing Institute of Technology Beijing 100081 China; ^2^ Institute of Advanced Technology Beijing Institute of Technology Jinan 250300 China; ^3^ Collaborative Innovation Center of Electric Vehicles in Beijing Beijing 100081 China

**Keywords:** electroplating, interface engineering, multifunctional 2D composite interface layer, zinc‐ion batteries

## Abstract

The practical application of AZIBs is hindered by problems such as dendrites and hydrogen evolution reactions caused by the thermodynamic instability of Zinc (Zn) metal. Modification of the Zn surface through interface engineering can effectively solve the above problems. Here, sulfonate‐derivatized graphene–boronene nanosheets (G&B‐S) composite interfacial layer is prepared to modulate the Zn plating/stripping and mitigates the side reactions with electrolyte through a simple and green electroplating method. Thanks to the electronegativity of the sulfonate groups, the G&B‐S interface promotes a dendrite‐free deposition behavior through a fast desolvation process and a uniform interfacial electric field mitigating the tip effect. Theoretical calculations and QCM‐D experiments confirmed the fast dynamic mechanism and excellent mechanical properties of the G&B‐S interfacial layer. By coupling the dynamics‐mechanics action, the G&B‐S@Zn symmetric battery is cycled for a long‐term of 1900 h at a high current density of 5 mA cm^−2^, with a low overpotential of ≈30 mV. Furthermore, when coupled with the LMO cathode, the LMO//G&B‐S@Zn cell also exhibits excellent performance, indicating the durability of the G&B‐S@Zn anode. Accordingly, this novel multifunctional interfacial layer offers a promising approach to significantly enhance the electrochemical performance of AZIBs.

## Graphical abstract

A simple and green electroplating method is employed to prepare the G&B‐S composite interfacial layer, regulating the Zn plating/stripping and mitigating the side reactions. Based on the dynamics‐mechanics coupling action, the G&B‐S@Zn symmetric battery can achieve long‐term cycling stability of 1900 h at 5 mA cm^−2^ with a small overpotential of ≈30 mV.

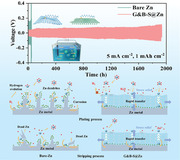

## 1 Introduction

The great enthusiasm for developing renewable energy has encouraged the development of high‐performance energy storage systems.^[^ ^1^ ^]^ To address safety issues and lithium resource constraints, a new energy storage system needs to be sought as a candidate for commercialized lithium‐ion batteries.^[^ ^2^, ^3^ ^]^ Aqueous zinc‐ion batteries (AZIBs) have attracted a lot of attention due to their low‐cost, high safety, and environmentalfriendly. Generally, Zinc (Zn) metal with high theoretical capacities (820 mAh g^−1^ and 5851 mAh cm^−3^) and abundant resources is a promising anode for AZIBs.^[^ ^4^, ^5^, ^6^ ^]^ Unfortunately, Zn dendrite growth and hydrogen evolution reaction (HER) inevitably encounter during repeated charging–discharging process, which may cause serious by‐effects such as low Coulombic efficiency (CE), capacity degradation, and even a short circuit.^[^ ^7^, ^8^, ^9^ ^]^

In response, a variety of strategies have been applied including interface engineering,^[^ ^10^, ^11^, ^12^, ^13^, ^14^, ^15^ ^]^ electrolyte optimization,^[^ ^16^, ^17^, ^18^ ^]^ 3D electrode construction,^[^ ^19^, ^20^, ^21^ ^]^ and so on, to achieve superior performance and good practicality. It is worth noting that Zn metal corrosion and HER directly determine the distribution of active sites on the Zn surface, resulting in uneven electron flux and further polarization. Both Zn dendrites and side reactions are closely related to the interfaces between the Zn anode and aqueous electrolyte. Amongst, the interface engineering is undoubtedly the most effective and direct method to solve these phenomena simultaneously.^[^ ^22^ ^]^

2D materials possess high specific surface area, tunable surface functional groups, and open 2D channels that facilitate ion transport.^[^ ^23^, ^24^ ^]^ Representatively, graphene and its derivatives have been employed as interfacial protective layers to enhance the corrosion resistance and diffusion kinetic of Zn anode.^[^ ^12^, ^25^ ^]^ With the in‐depth research on graphene, more and more atomically thin materials have been successfully derived, such as germanene, phosphorene, and silicene.^[^ ^26^, ^27^, ^28^ ^]^ As a neighbor of carbon elements, boron shares many of the same characteristics as carbon. Boronene, on the other hand, is a new 2D form of boron element, which has attracted great interest due to its extraordinary properties, including anisotropic metallic behavior, and superconductivity, as well as enhanced mechanical properties and flexibility.^[^ ^29^, ^30^ ^]^ To evaluate the suitability of boronene in energy storage, it has been successfully used as an electrode material or anchoring material, showing better properties such as higher capacity, low diffusion energy barrier, and high electrical conductivity.^[^ ^31^, ^32^, ^33^, ^34^, ^35^ ^]^ Importantly, boronene can be used as a Lewis acid, which has a strong reactivity that can enhance surface interactions.^[^ ^36^ ^]^ However, it is worth noting that as of now, the utilization of borophene in electrode interface modification has not been realized.

Nevertheless, a single 2D material can barely meet the multiple requirements of a high‐performance interfacial layer such as high ionic conductivity, excellent mechanical performance, strong stability, and so on.^[^ ^22^, ^37^ ^]^ Composite or modification opens up new possibilities for adjusting the physical and chemical properties of the 2D materials. Therefore, more 2D composites should be designed to attain the multifunctional interfacial layers.

Herein, we report a multifunctional graphene/boronene (G&B) 2D composite as an interfacial regulation layer prepared by simple, green, and controllable electroplating method. The dodecyl sulfonate anionic surfactant introduced into the electroplating process that further boosts the functionalization of G&B, affording a stronger electronegativity of sulfonate‐derivatized G&B (G&B‐S) protective layer. The electrostatic assembly of graphene and boronene reduces the reactivity between the interface and electrolyte, while enhancing the attraction to Zn atoms. Based on the G&B‐S@Zn anode, the mitigation of the tip effect produces a flat deposition morphology, and the G&B‐S interfacial layer with better elasticity can inhibit the growth of Zn dendrites even with the long‐term plating/stripping process. Based on the synergistic effect of enhanced dynamics and mechanics in interface, the G&B‐S@Zn electrode exhibits stable durability, particularly under the high current density. The benefits of the G&B‐S interfacial layer are also evident in the LMO//G&B‐S@Zn cell.

## 2 Results and Discussion

**Figure 1:**
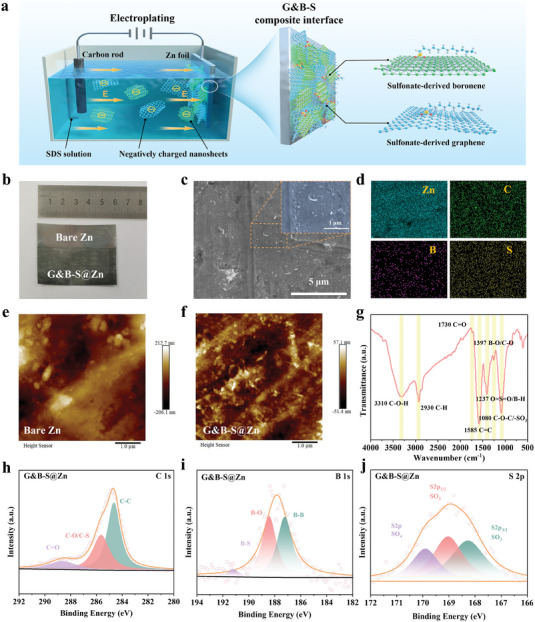
a) Schematic illustration of fabricated G&B‐S interface layer by electroplating method. b) Optical image of the G&B‐S@Zn. c,d) SEM images of G&B‐S@Zn sample and the corresponding EDS mapping of elemental Zn, C, B, and S. e,f) AFM images of bare Zn and G&B‐S@Zn. g) FT‐IR spectra of G&B‐S@Zn. h–j) C 1s, B 1s, S 2p XPS spectrum of G&B‐S@Zn sample.

To obtain the G&B‐S decorated Zn electrode, a green and simple electroplating approach was applied to construct an interfacial layer as shown in **Figure** **1**a and Figure S1 (Supporting Information). Monolayer graphene and boronene nanosheets with similar sizes (Figures S2 and S3, Supporting Information) were selected to configure the electroplating solution using sodium dodecyl sulfate (SDS) as anionic surfactant. The negatively charged graphene and boronene nanosheets move toward the Zn surface driven by an applied electric field to form a uniform interfacial layer with an alternating stack. After drying at 80 °C for 12 h to enhance the interaction of the electroplated film to the Zn foil, the Zn substrate shows a uniform black coating of the G&B‐S layer (Figure 1b). The scanning electron microscopy (SEM) image of the polished Zn foil with a rough surface is shown in Figure S4 (Supporting Information), whereas the G&B‐S @Zn exhibits a flat morphology (Figure 1c). The corresponding energy‐dispersive X‐ray spectroscopy (EDS) mapping confirms the homogeneous distribution of C, B, and S elements, ensuring the successful formation of the functional group‐modified composite film (Figure 1d; Figure S5, Supporting Information). Meanwhile, atomic force microscopy (AFM) was employed to characterize the surface microstructure of the G&B‐S interfacial layer. The AFM image of bare Zn shows a higher average height difference (Figure 1e), while the G&B‐S @Zn exhibits a flat feature of graphene and boronene nanosheets (Figure 1f).

Fourier‐transform infrared (FT‐IR) spectroscopy was used to identify the surface functional groups of the G&B‐S layer. The characteristic peaks of the surface groups (such as C─O, C─O─C, B─O, and B─H bonds) can be clearly observed, indicating the successful self‐assembly of graphene with boronene. Additionally, the ─SO_3_H group in SDS produces characteristic bands corresponding to O═S═O and SO_3_− stretching, which reveals that the sulfonic acid group was anchored on the G&B surface during the electroplating process (as shown in Figure 1g). Inconsistent with the FT‐IR results, the X‐ray photoelectron spectroscopy (XPS) confirms the existence of C, B, and S elements in the G&B‐S interfacial layers (Figure S6, Supporting Information). As shown in XPS of deconvoluted C 1s (Figure 1h) and B 1s (Figure 1i) peaks, the C─O/C─S (285.7 eV) and C═O bonds (288.7 eV), as well as B─B (187.2 eV), B─O (188.5 eV), and B─S (191.2 eV) bonds can be observed to verified the existence of graphene and boronene, respectively. For the S 2p spectrum in Figure 1j, three major components are identified to be successful modifications of the ─SO_3_H group.^[^ ^38^ ^]^ Obviously, both FT‐IR and XPS analysis proved the successful composition with the G&B interfacial layer and modification of negatively charged sulfonic groups.

**Figure 2:**
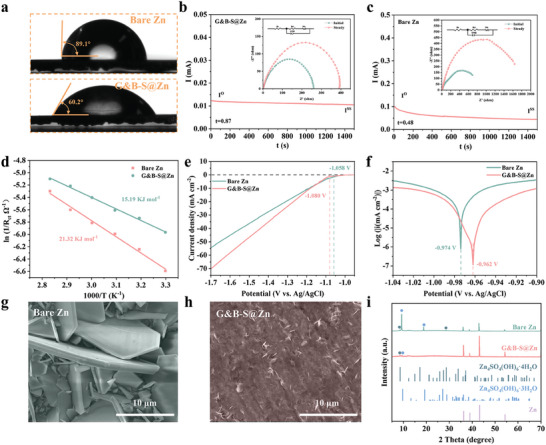
a) Contact angles of bare Zn and G&B‐S@Zn electrodes with 2 m ZnSO4 electrolyte. Zn^2+^ transference number characterization of b) bare Zn and c) G&B‐S@Zn. d) Arrhenius curves and comparison of activation energies of bare Zn and G&B‐S@Zn. e) Linear sweep voltammetry curves and f) Tafel curves of bare Zn and G&B‐S@Zn. g,h) SEM images and i) corresponding XRD patterns of bare Zn and G&B‐S@Zn immersed in 2 m ZnSO4 aqueous solution for 10 days.

As displayed in **Figure** **2**a, the interfacial compatibility of the prepared G&B‐S@Zn electrode with 2 *m* ZnSO_4_ electrolyte was further tested by contact angle measurement. It is obviously observed that the G&B‐S@Zn electrode exhibits a better wettability at 89.1^°^ than the bare Zn electrode at 60.2^°^, indicating a stronger interaction between the aqueous electrolyte and the G&B‐S layer attributed to the hydrophilic sulfonic groups. The discrepant wettability between electrolyte and bare Zn electrode will lead to uneven surface charge distribution and disordered Zn deposition, while close interfacial contact can ensure a uniform surface local current. Thermodynamically considered, the enhanced wettability further reduces wetting free energy at the electrolyte‐electrode interfaces, indicating a rapid ion transfer between the electrolyte and G&B‐S@Zn. This speculation can be verified by the high Zn^2+^ transference number of 0.87 (Figure 2b). Moreover, the negatively charged interfacial layer can enhance the diffusion kinetics of Zn^2+^ due to the strong electrostatic interaction, which relieves the interfacial concentration difference and promotes uniform Zn deposition. These interactions ensure the swift migration of Zn ions and the potential for early desolvation, effectively mitigating water‐induced side reactions. In contrast, the low Zn^2+^ transference number of 0.48 in a bare Zn symmetric battery can be ascribed to the slow migration of solvated Zn^2+^ in an aqueous solution that forms an imbalance between electrons and ions at interfaces (Figure 2c). Hence, a large Zn^2+^ concentration gradient near the Zn metal surfaces, causes the dendrites growth consequently. Figure S7a,b (Supporting Information) show the electrochemical impedance spectroscopy (EIS) of bare Zn and G&B‐S@Zn electrodes at different temperatures. The charge‐transfer resistance (*R*_ct_) value of the G&B‐S@Zn electrode is considerably lower than that of the bare Zn electrode at all temperatures, which confirms the improved charge‐transfer capability in the G&B‐S layer. Meanwhile, the corresponding activation energy of G&B‐S@Zn was calculated by the Arrhenius equation^[^ ^17^ ^]^ as 15.19 kJ mol^−1^, while that of bare Zn was 21.32 kJ mol^−1^, the lower activation energy further demonstrating that the G&B‐S layer accelerates the desolvation process and transfer rate of Zn^2+^ (Figure 2d).

To verify the hydrogen evolution inhibition and corrosion resistance of the G&B‐S@Zn electrode, linear scanning voltammetry (LSV) and tafel curves were employed. As shown in Figure 2e,f, the G&B‐S@Zn electrode exhibits a lower hydrogen evolution potential (−1.080 V) than that of bare Zn (−1.058 V) and a higher corrosion initiation potential (−0.962 V) compared to that of bare Zn (−0.974 V), suggesting that the G&B‐S layer effectively suppressed various side reactions.^[^ ^39^ ^]^ To further illustrate the anti‐water‐induced corrosion properties of G&B‐S layer, bare Zn and G&B‐S@Zn were immersed in 2 m ZnSO_4_ aqueous solution for several days. From Figure 2g and Figure S8a (Supporting Information), it can be seen that the surface of bare Zn metal shows obvious flaky corrosion products. While the morphology of G&B‐S@Zn remains unchanged with the increase of soaking days for 3, 5, 7, and 10 days, respectively (Figure 2h; Figure S8b, Supporting Information). Furthermore, when immersed in 1 m ZnSO_4_ and 1 m KOH solutions, G&B‐S@Zn exhibited equally impressive stability (Figure S9, Supporting Information). Moreover, the corresponding XRD patterns of G&B‐S@Zn after 10 days of immersion display almost no impurities, indicating that the G&B‐S layer can enhance the thermodynamic stability of Zn metal in a corrosion environment. In contrast, the XRD patterns of bare Zn clearly show the sharp diffraction peaks of Zn_4_SO_4_(OH)_4_−3H_2_O and Zn_4_SO_4_(OH)_4_−4H_2_O, which can be assigned to the by‐products of basic Zn sulfate, as shown in Figure 2i. The severe passivation of bare Zn may block the transport pathways of Zn^2+^.

**Figure 3:**
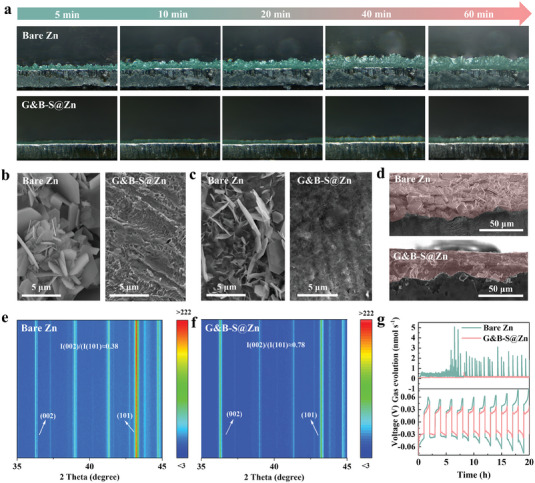
a) In situ optical microscopy visualization of Zn deposition on bare Zn and G&B‐S @Zn at 5  mA cm^−2^ for 60 min. b) The deposited morphology of bare Zn and G&B‐S@Zn at 1 mA cm^−2^ with 1 mAh cm^−2^. c) The top view and d) side view of bare Zn and G&B‐S@Zn electrodes after 100 h at 1 mA cm^−2^ with a capacity of 1 mAh cm^−2^. e,f) In situ XRD profiles and g) in situ DEMS profiles of bare Zn and G&B‐S@Zn symmetric batteries.

To visually investigate the effect of the G&B‐S interfacial layer on the suppression of Zn dendrites, an in situ optical microscopy technique was performed to study the Zn deposition behavior. As shown in **Figure** **3**a, as expected the surface of the bare Zn electrode randomly appeared uneven Zn plating with tiny protrusions, which gradually gathered into clusters and became many visible dendrites over time due to uneven electric field distribution. In stark contrast, the G&B‐S@Zn induced uniform nucleation sites to form a flat surface without dendrites after 60 min of Zn deposition. The excellent performance can be attributed to the strong interaction force between the enriched active sites in the G&B‐S interface and Zn^2+^ in an electrolyte, which induces uniform deposition of Zn^2+^ toward the electrode. Therefore, the G&B‐S layer can be employed as a rectifier to alleviate the inhomogeneity of local charge density distribution, thereby inhibiting the nucleation and growth of local Zn dendrites. Furthermore, according to the SEM images, the dense upright Zn flakes and disordered Zn clusters are presented on the bare Zn foil after deposition with a real capacity of 1 mAh cm^−2^ (Figure 3b), which may lead to short circuits under unrestricted dendritic growth. While the G&B‐S@Zn exhibits a relatively flat surface and fully covered protective layer. Additionally, the surface and side morphology of each electrode after 100 h is displayed in Figure 3c,d. A large amounts of Zn dendrites and by‐products can be clearly seen on the surface of the bare Zn electrode after long‐term cycling, and the deposited Zn formed a porous layer with greater thickness. With the help of the G&B‐S layer, the G&B‐S@Zn electrode surface remained smooth without significant changes compared to the initial state, and the cross‐sectional image shows dramatically reduced dendrite growth.

In‐situ XRD test was used to further investigate the Zn deposition process (Figure 3e,f) and the corresponding electrochemical charge–discharge curves are shown in Figure S10 (Supporting Information). The intensity of Zn (101) characteristic peak of the bare Zn electrode is stronger than that of the G&B‐S@Zn electrode, but the intensity of Zn (002) characteristic peak is weaker on the latter. It is evident that the I(002)/I(101) value (0.78) of the G&B‐S@Zn electrode is higher than that of the bare Zn electrode (0.38), signifying a greater proportion of Zn(002) relative to Zn(101). This result indicates that the metallic Zn was deposited on the G&B‐S@Zn electrode mainly following the orientation of the Zn (002) crystal plane, which is less prone to grow into dendrites.^[^ ^40^, ^41^ ^]^ Whereas, bare Zn electrode exhibited the orientation of the Zn (101) crystal plane for Zn deposition, corresponding to a high risk of dendritic growth. To better understand the mechanism of G&B‐S interfacial layer for inhibiting HER, in situ measurements of hydrogen evolution rate (*R*_H_) by differential electrochemical mass spectrometry (DEMS) were performed. The *R*_H_ of the bare Zn symmetric battery gradually increased from 0.65 nmol s^−1^ to as high as 5 nmol s^−1^ after 5 cycles, as shown in Figure 3g. The increase in hydrogen flux can be attributed to the partial oxidation of Zn. Furthermore, the corrosion passivation layer impedes the diffusion of Zn^2+^, resulting in the increase of the polarization voltage. For comparison, the *R*_H_ of the G&B‐S@Zn symmetric battery almost remained at a low rate of 0.2 nmol s^−1^, implying that hydrogen release was suppressed during cycling. In brief, the multifunctional composite interfacial layer not only induces the formation of Zn (002) preferential deposition without dendritic growth, but also homogenizes the ion flux to attain low polarization voltage. Meanwhile, the G&B‐S layer can reduce the reaction activity between water molecules and metallic Zn, and then mitigate the HER.

**Figure 4:**
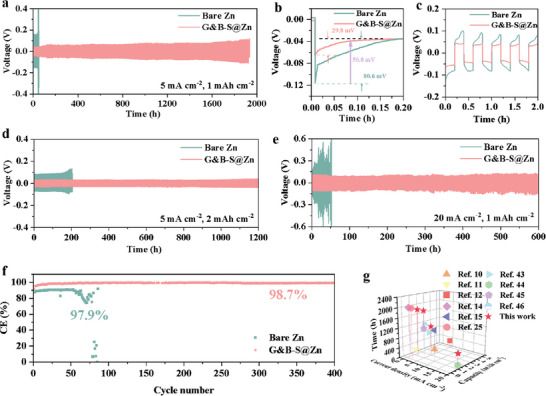
a–c)Galvanostatic cycling performance of bare Zn and G&B‐S@Zn symmetric batteries at 5 mA cm^−2^ for 1 mAh cm^−2^, d) 5 mA cm^−2^ for 2 mAh cm^−2^ and e) 20 mA cm^−2^ for 1 mAh cm^−2^. f) The CE measurements of bare Zn//Cu and G&B‐S@Zn//Cu at 1 mA cm^−2^ with a capacity of 1 mAh cm^−2^. g) The Comparison of cycling performance between this work and other previous reports.

Subsequently, to evaluate the reversibility of the Zn plating/stripping process in the presence of the G&B‐S layer, the Zn//Zn and G&B‐S@Zn//G&B‐S@Zn symmetric batteries were assembled. As expected, the G&B‐S @Zn electrode exhibited significantly improved cycling stability. The G&B‐S@Zn symmetric battery delivered an ultra‐long cycling life over 1900 h at 2 mA cm^−2^ with an area capacity of 1 mAh cm^−2^, while the bare Zn symmetric battery showed an uncontrollable short‐circuiting caused by dendritic growth at less than 100 h (Figure S11, Supporting Information). Additionally, the G&B‐S@Zn electrode operated stably over 1900 h at a high current density of 5 mA cm^−2^ (**Figure** **4**a). Meanwhile, the overpotential of the G&B‐S@Zn electrode (≈30 mV) is much lower than that of bare Zn (≈81 mV) (Figure 4b,c), which can be attributed to the enhanced Zn^2+^ migration. In addition, the *R*_ct_ value of the symmetrical battery with G&B‐S@Zn electrode under various cycles are significantly lower than that of the bare symmetrical battery, once again confirming the enhancement of the interface kinetic by G&B‐S layer (Figure S12, Supporting Information). When the capacity increased to 2 mAh cm^−2^, the long‐term cycling life of the G&B‐S@Zn symmetric battery exceeded 1200 h, which is better than that of the bare Zn symmetric battery (Figure 4d) at the same current density of 5 mA cm^−2^, indicating that the G&B‐S layer contributes rapid ion transport and high corrosion resistance to ensure reversible plating/stripping of metallic Zn. Even at the elevated current density of 20 mA cm^−2^, the G&B‐S@Zn symmetric battery can still cycle over 600 h with a lower polarization voltage (112 mV), thanks to the enhanced kinetic response mechanism of the G&B‐S@Zn electrode (Figure 4e).

It is clear that the bare Zn electrodes succumb to larger voltage fluctuations during the repetitive plating/stripping process, exhibiting greater voltage hysteresis of 73.5, 75.2, 90.4, 127.2, and 232 mV at current densities of 1, 2, 5, 10 and 20 mA cm^−2^ (Figures S13 and S14, Supporting Information), respectively. The G&B‐S@Zn electrodes exhibit a flat and stable voltage plateau at each current density, corresponding to lower polarization voltages of 34.2, 39.7, 52, 70.6, and 122.2 mV. The high‐rate performance can be expressed by the enhanced ion migration kinetics at the electrode–electrolyte interfaces. The positive effect of the G&B‐S layer on the high CE was also investigated. As shown in Figure 4f, the CE of the bare Zn//Cu half battery rapidly fluctuates and continuously decreases after ≈60 cycles with a capacity of 1 mAh cm^−2^ at 1 mA cm^−2^, which is caused by the dendrite‐induced short circuit. While the average CE of the G&B‐S@Zn//Cu half battery remains ≈98.7% after 400 cycles, indicating excellent reversibility of Zn plating/stripping after a short activation and stabilization process. Cyclic voltammetry (CV) tests also further confirm the higher electrochemical reactivity and faster reaction kinetics, showing higher current density and lower overpotential on G&B‐S@Zn electrode (Figure S15, Supporting Information). Eventually, Figure 4g compares the electrochemical properties with those reported previously, which illustrates the outstanding rate and cycling performances for G&B‐S modified interface engineering.^[^ ^42^, ^43^, ^44^, ^45^ ^]^ Subsequently a thorough comparison of several critical electrochemical parameters in symmetric cells with various interface‐modified Zn anodes, including the synthesis method, current density, areal capacity, cycle life, and average voltage hysteresis, as demonstrated in Table S1 (Supporting Information). Hence, the outstanding performance achieved by the G&B‐S@Zn electrode has been confirmed.

**Figure 5:**
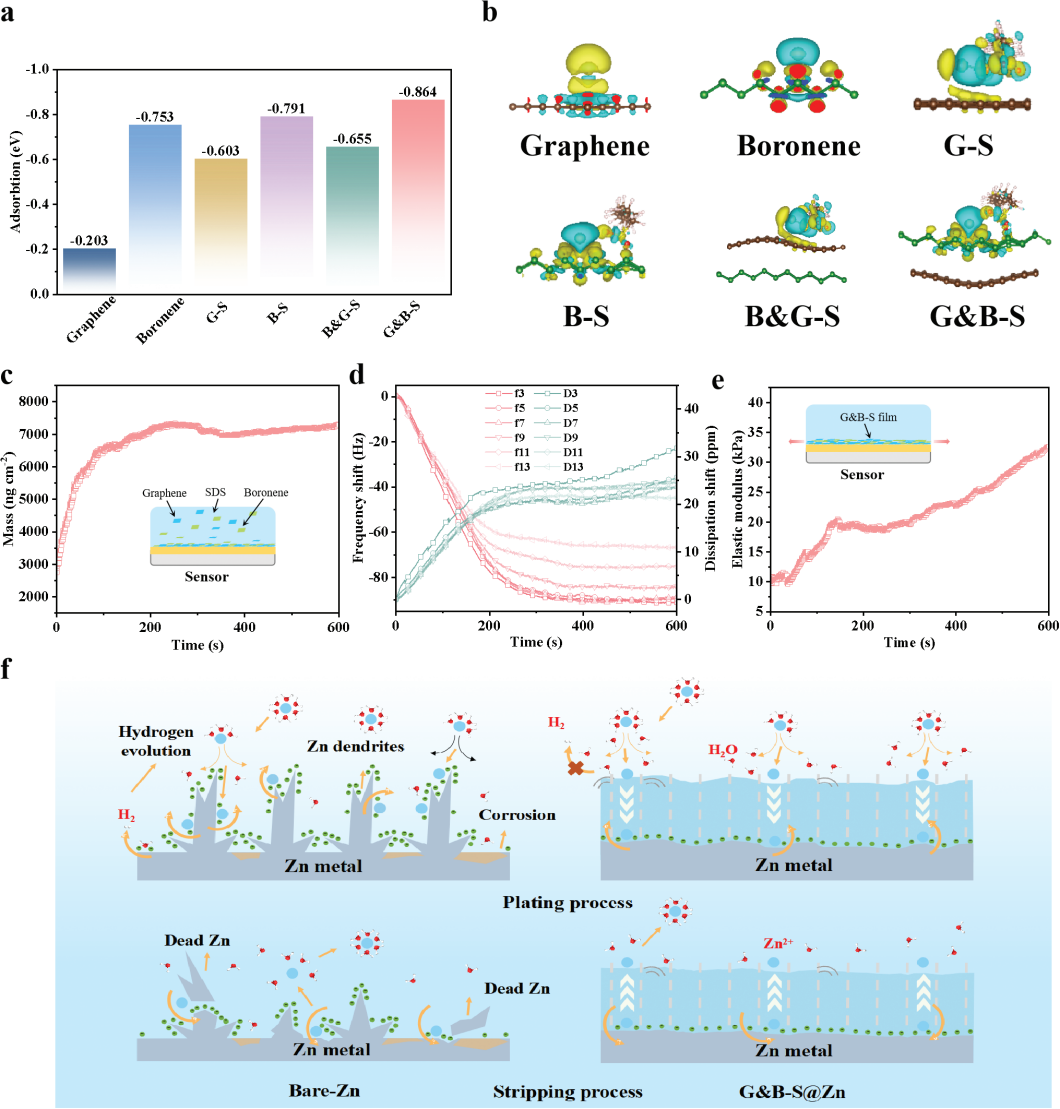
a) Summary of the calculated adsorption energies of Zn atom adsorbed on pure graphene, boronene, G‐S, B‐S, B&G‐S, and G&B‐S. b) Isosurfaces of charge density difference of Zn adsorbed on the surface of graphene, boronene, G‐S, B‐S, B&G‐S, and G&B‐S, the loss of electrons is indicated in blue, and the gain of electrons is indicated in yellow. c) Mass, d) frequency shift, and e) elasticity modulus of the crystal‐adsorbed G&B‐S layer in QCM‐D measurement. f) Schematic diagram illustrating the plating/stripping process of bare Zn and G&B‐S@Zn electrodes.

The diffusion behavior of Zn was investigated by density functional theoretical calculation to further verify the influence of the G&B‐S interface on the deposition process and electrochemical properties of Zn anode. As shown in Figure S16 (Supporting Information), the high‐resolution TEM image of boronene nanosheets determines the crystal structure model.^[^ ^46^ ^]^ The adsorption configurations of Zn atoms on pure graphene, pure boronene, sulfonate‐derived graphene (G‐S), sulfonate‐derived boronene (B‐S), and sulfonate‐derived boronene–graphene (B&G‐S) / graphene–boronene (G&B‐S) composite interface structures are shown in Figure S17 (Supporting Information). Based on this, **Figure** **5**a compares the adsorption energies of Zn atoms on the surfaces of pure graphene, boronene, G‐S, B‐S, B&G‐S, G&B‐S, respectively. The adsorption energy of the Zn atom is the weakest on pure graphene surface, and the strongest on the sulfonate‐derived boronene surface in G&B‐S, where the adsorption energy was enhanced from −0.203 to −0.864 eV. According to the calculation results, it can be inferred that the high interfacial adsorption energy can be attributed to the strong interaction between the Zn atom and the G&B composite, while the Lewis acid of boronene and the sulfonic acid groups further promote this interaction. This fact is also confirmed by the differential charge density. Figure 5b presents the differential charge densities of these models to explain the charge transfer during the composite and adsorption processes. In the Zn deposition process, the relatively low charge density of boronene causes a large amount of charge transfer from the Zn atom to boronene, which is not obvious in graphene. And then the oxygen atom in the sulfonic acid group of SDS possesses a strong electron‐absorbing effect, which helps to reduce the charge distribution on the boronene surface. Therefore, the tighter connection between the Zn atom and boronene can be attained by deep charge exchange. Moreover, the introduction of a composite structure can further enhance the adsorption ability of Zn atom according to the adaptive conduction mode. Due to the strong interaction and improved kinetics, the concentration gradient of Zn atoms at the G&B‐S surface is reduced to avoid the formation of dendrites. Correspondingly, the high electrical conductivity of the composite can achieve the uniform distribution of electrons at the Zn anode surface. Furthermore, the electrochemical performance of G‐S@Zn//G‐S@Zn and B‐S@Zn//B‐S@Zn symmetric batteries lend further support to these computational findings. At a high current density of 20 mA cm^−2^, the polarization voltages for bare Zn, G‐S@Zn, B‐S@Zn, and G&B‐S@Zn symmetric batteries were 394, 126, 114, and 92 mV (Figure S18, Supporting Information), respectively. This observed trend aligns with the variation in adsorption energies of different components, reinforcing the excellent diffusion behavior of Zn at the G&B‐S interface.

To further illustrate the variation in the interfacial stresses of the interfacial layer to withstand during the continuous plating/stripping, the quartz crystal microbalance with dissipation monitoring (QCM‐D) was employed to verify the mechanical properties of the G&B‐S film.^[^ ^47^, ^48^ ^]^ First, Figure 5c shows the interfacial film mass during the electroplating obtained by the QCM as a function of time, where the QCM mass oscillates in the presence of an elevated tendency before remaining stable. It can be inferred that the electronegative nanosheets are successfully adsorbed onto the sensor, forming a stable interfacial film under the effect of a constant current. In the interfacial layer adsorption phase, the frequency shifted (Δ*f*) decreased and dissipation shifted (ΔD) increased, and then gradually stabilized with time, indicating the saturation of the G&B‐S coverage on the crystal surface (Figure 5d). It should be noted that the difference between Δ*f* and Δ*D* for each frequency shift is large, suggesting the formation of a relatively soft G&B‐S interface film. Further plotting of dissipation with frequency allows tracking the lower Δ*f*/Δ*D* value of energy dissipation per unit mass of the crystal adsorption layer, expressing the formation of film with soft and flow on the QCM‐D sensor.^[^ ^49^ ^]^ As shown in Figure S19 (Supporting Information), the Δ*f*/Δ*D* value gradually decreased until it stabilized, which is an interfacial film system with loose and soft. In other words, with the adsorption of the G&B‐S interfacial film, the crystal adsorption layer per unit mass can cause more energy dissipation, resulting in an adhesion layer with superior flexibility. With such a high elasticity property, the G&B‐S interfacial layer can thus withstand the long‐term plating/stripping of metallic Zn. Moreover, the G&B‐S interfacial layer with a higher elasticity (Figure 5e), which can thus moderate changes in interfacial stresses during long‐term plating/stripping of metallic Zn.

Based on the above analysis, the mechanism of the coupling of interfacial film dynamics and mechanics is proposed (Figure 5f). The G&B‐S interface effectively mitigates hydrogenic corrosion by accelerating the desolvation process through the strong interaction between the established electronegative G&B‐S interface and Zn^2+^, while also physically blocking the Zn electrode from water. Additionally, during the plating process, the G&B‐S layer ensures the rapid transport of Zn^2+^ and reduces the concentration gradient at the interfaces, mitigating the tip effect and preventing dendrite formation. The strong interaction of S‐graphene and S‐boronene complexes with Zn atoms enhances the kinetic reaction mechanism, leading to the deposition of the metallic Zn (002) crystal plane. The G&B‐S acts as a guiding layer, controlling Zn deposition and mitigating the formation of Zn dendrites and “dead” Zn. Furthermore, the superior flexible nature and excellent mechanical properties of the G&B‐S layer moderate interfacial stress changes during the electrochemical reaction process, ensuring the long‐term cycling stability of the Zn anode. In contrast, the bare Zn anode induces the formation of Zn dendrites due to the tip effect, resulting in a significant expansion of the electrode volume during the plating/stripping process and gradual failure over long‐term cycling. In conclusion, the theoretical analyses strongly justify the outstanding electrochemical behavior of the G&B‐S@Zn electrode in the experimental measurements explained above.

**Figure 6:**
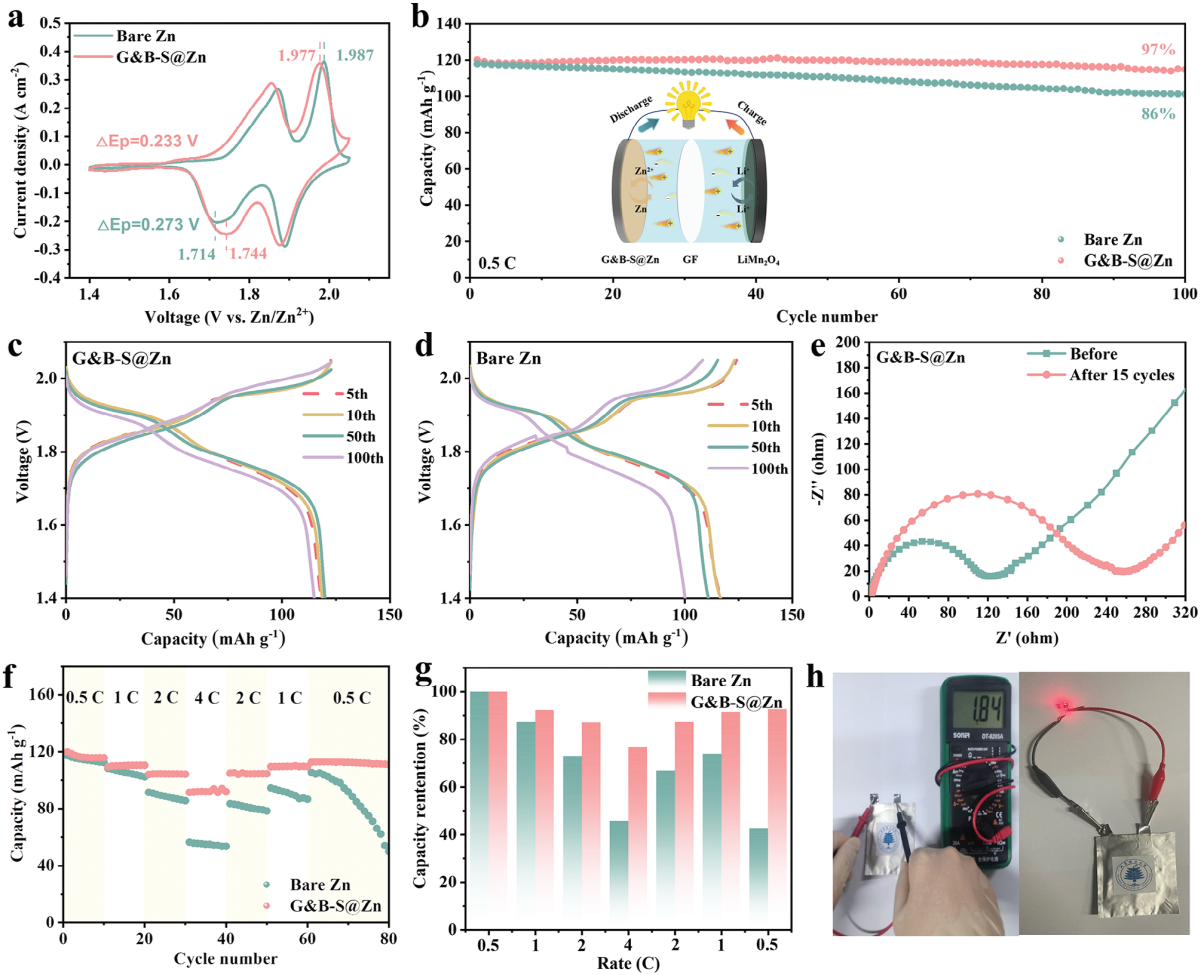
a) CV curves of LMO//bare Zn and LMO//G&B‐S@Zn cells. b) Cycling performance of LMO//bare Zn and LMO//G&B‐S@Zn cells at 0.5C. c) Voltage profiles of the LMO//bare Zn and d) the LMO//G&B‐S@Zn at different cycles. e) Nyquist plots of LMO//G&B‐S@Zn cell before and after cycling. f) Rate performance of LMO//bare Zn and LMO//G&B‐S@Zn at different rates of 0.5C, 1C, 2C, and 4C. g) The comparison of corresponding capacity retention. h) Digital images displaying the working states of LMO//G&B‐S@Zn pouch cell to power an LED indicator.

Lithium manganese oxide (LMO) //G&B‐S@Zn cell is assembled to demonstrate the feasibility of G&B‐S@Zn for practical applications. From the results of the CV curves shown in **Figure** **6**a, the potential differences between the redox peaks of the LMO//G&B‐S@Zn cell measured at the scan rate of 0.1 mV s^−1^ are lower than that of the LMO//bare Zn cell, revealing the reduced polarization and the accelerated reaction kinetics in the former. Hence, a long‐term cycling performance of LMO//G&B‐S@Zn cell can be achieved at 0.5C, as depicted in Figure 6b, 97% of the initial capacity is still retained after 100 cycles. Conversely, the pristine LMO//bare Zn cell exhibits severe degradation, retaining only 86% of its initial capacity after the same cycles. The superior performance of the LMO//G&B‐S@Zn cell is also analyzed by comparing the corresponding charge–discharge capacity–voltage curves with the LMO//bare Zn (Figure 6c,d). The capacity of LMO//bare Zn cell decreased significantly after 100 cycles, while the cell with G&B‐S@Zn anode delivers a stable cyclability. The EIS reveals that both the initial and cycled resistances of cells. As shown in Figure 6e and Figure S20 (Supporting Information), the G&B‐S@Zn‐based cell provides a *R*_ct_ value of 200 Ω before cycling, which is much lower than that of the control battery (450 Ω). Such a low resistance facilitates fast ion transfer at interfaces. Obviously, the resistance of LMO//bare Zn increased significantly after 15 cycles, which may be due to the formation of a corrosion passivation layer on the Zn anode. As for the rate performance (Figure 6f,g), it is clear that the high‐rate capacity of the modified system can also be greatly improved. The discharge capacities of LMO//G&B‐S@Zn cell at increased rates of 0.5, 1, 2, and 4C are 248.5, 228.5, 220.2, and 201 mAh g^−1^, respectively. Notably, LMO//G&B‐S@Zn exhibits better reversibility, while the LMO//bare Zn shows a sharp capacity decay after a rapid charging–discharging process. Furthermore, a pouch cell with an open‐circuit voltage of 1.84 V was assembled by applying the large‐scale G&B‐S@Zn electrode, which was able to light up a light‐emitting diode (LED) indicator stably (Figure 6h). On this basis, it was demonstrated that the designed G&B‐S@Zn anode could be applied in a practical scenario device, thus showing great potential for the development of high‐performance AZIBs.

## 3 Conclusion

In summary, we designed a multifunctional G&B‐S interfacial layer for the Zn anode by a simple green electroplating preparation method. Under the mechanism of coupled dynamical and mechanical effects, this interfacial layer can accelerate the desolvation of Zn ions and ensure the rapid migration of ions. In situ tests illustrate that the interfacial layer help to induce the exposure of more Zn (002) crystal plane that is less prone to grow into dendrites while suppressing HER. Synergistically, the G&B‐S@Zn anode enables a considerably enlarged cycle of ≈2000 h at a high current density of 5 mA cm^−2^ with a capacity of 1 mAh cm^−2^, and the LMO//G&B‐S@Zn cell further validates the usefulness of the protective layer. This work illustrates the significance of a rationally designed advanced protective coating for Zn metal, which may also be applicable to other metal anodes.

## 4 Experimental Section

### Synthesis of G&B‐S@Zn Anode and LMO Cathode

Graphene aqueous dispersion (1 mg mL^−1^), boronene aqueous dispersion (1 mg mL^−1^), and sodium dodecyl sulfate aqueous solution (0.0005 m) were mixed in a volume ratio of 1:1:3, and citric acid/sodium citrate was used to adjust the pH of mixed solution to ≈5. The electroplating solution was obtained by continuous magnetic stirring evenly. It should be noted that the addition of the SDS was intended to facilitate the movement of graphene and boronene nanosheets toward the Zn electrode surface along the applied electric field during the electroplating process.

Then, Zn foil (100 µm) was used as the working electrode, and carbon rod was used as the counter electrode to form the two‐electrode system. The electroplating was carried out by the constant current method (0.0005 A) for 15 min to obtain the G&B‐S@Zn anode. It should be noted that the unadsorbed plating solution on the surface of the Zn foil was removed by rinsing several times with deionized water. Finally, the electroplated Zn foil was dried at 60 °C for 24 h to enhance the bonding of the interfacial layer with metallic Zn.

To prepare the LMO cathode, 70 wt.% commercial LMO, 20 wt.% Super P, and 10 wt.% PVDF in NMP solvent were ground for ≈40 min, and the homogeneous slurry was subsequently coated to the titanium foil. Then, the prepared electrodes were vacuum dried in an oven at 80 °C for 12 h.

### Material Characterization

The morphology of the prepared G&B‐S@Zn and bare Zn electrodes were characterized by SEM (FEI XL30 Sirion) at an accelerating voltage of 10 kV. The morphology of Zn deposition and after cycling were also obtained. EDS elemental maps were used to define the distribution of elements. The microstructures of the products were determined by TEM (FEI Tecnai G2 F30). AFM (Bruker Multimode 8 with a Nanoscope V controller) was used to further understand the morphology of the G&B‐S@Zn and bare Zn. Bruker Alpha FT‐IR spectrometer (ATR‐Ge, 1000–4000 cm^−1^) were recorded on FT‐IR spectra. XPS measurements characterized the chemical composition of the G&B‐S@Zn sample on a spectrometer (PerkinElmer PHI 1600 ESCA) with Al Kα radiation (hv = 1486.6 eV). XRD (Bruker D8 Advance) with CuKα radiation range 5°–70°. The contact angle test (OCA20, Dataphysics, Germany) was characterized the wettability between the electrolyte and the G&B‐S interfacial layer. In situ DEMS (Hidden HPR‐40) was used to monitor the hydrogen evolution rate (*R*_H_) during the plating/stripping of symmetric cells. In situ XRD test was used to investigate the Zn deposition process. QCM‐D (Biolin Scientific) was employed to characterize the mechanical properties of the G&B‐S film.

### Electrochemical Characterization

The type of symmetric cells were assembled with two identical electrodes that G&B‐S@Zn or bare Zn. Meanwhile, glass fiber was investigated as the separator and 2.0 m ZnSO_4_ as the electrolyte. The Cu foil was directly used as the working electrode, the prepared G&B‐S@Zn or bare Zn was used as both the counter and reference electrode to assemble the half coin cell. For the LMO//G&B‐S@Zn or LMO//bare Zn cell, 1.0 m ZnSO_4_ and 1.0 m Li_2_SO_4_ with 0.1 m MnSO_4_ flooded aqueous solution to apply as the electrolyte.

To assemble the LMO//G&B‐S@Zn pouch cell, an LMO cathode (6 × 5 cm^−2^) and a G&B‐S@Zn anode were coupled. A pouch cell was constructed using a 1.0 m ZnSO_4_ and 1.0 m Li_2_SO_4_ with 0.1 m MnSO_4_ flooded aqueous solution as the electrolyte. All electrochemical measurements were conducted with glass fiber separators.

The Neware cell tester was used to examine the long‐cycle performance of all cells as well as the electrochemical deposition behavior of Zn metal. The LSV, Tafel, CV, and EIS measurement were conducted using a CHI 660e electrochemical workstation. Zn ion transference number was measured on the bare Zn and G&B‐S@Zn symmetric cells by a CHI 660e electrochemical workstation.

### Calculation Method

All density functional theory (DFT) calculations within the generalized gradient approximation (GGA) using the Perdew–Burke–Ernzerhof (PBE) functional were performed employing the Vienna Ab initio Simulation Package (VASP).

The projected augmented wave (PAW) potentials were chosen to describe the ion nuclei and the valence electrons were taken into account using a plane wave basis set with a kinetic energy cutoff of 400 eV. The DFT‐D3 empirical correction method was employed to describe van der Waals interactions. Geometry optimizations were performed with the force convergency smaller than 0.05 eV Å^−1^ and all atoms are relaxed in all the calculations.

The adsorption energy (Ea) was calculated by the equation:

(1) $$\mathrm{Ea}=E\left(\mathrm{total}\right)-E\left(\mathrm{slab}\right)-E\left(\mathrm{Zn}\right)$$

where *E*(total) and *E*(slab) correspond, respectively, to the total energy of the surface slab with and without Zn adsorption, and *E*(Zn) is the energy of single Zn.

## Conflict of Interest

The authors declare no conflict of interest

## Supporting information

Supporting Information

## Data Availability

The data that support the findings of this study are available from the corresponding author upon reasonable request.
