# Supplementary material for: Interface Engineering with Dynamics‐Mechanics Coupling for Highly Reactive and Reversible Aqueous Zinc‐Ion Batteries
Source: Adv Sci (Weinh). 2023 Dec 2;10(36):2306656. doi: 10.1002/advs.202306656 (PMC10754080; doi:10.1002/advs.202306656)
Supplement: Supplementary file 1 — Supporting Information [file ADVS-10-2306656-s001.pdf]

ADVANCED SCIENCE
Open Access

Supporting Information

for *Adv. Sci.*, DOI 10.1002/advs.202306656

Interface Engineering with Dynamics-Mechanics Coupling for Highly Reactive and Reversible Aqueous Zinc-Ion Batteries

Qianqian Meng, Xiaoyu Jin, Nuo Chen, Anbin Zhou, Huirong Wang, Ning Zhang, Zhihang Song, Yongxin Huang, Li Li, Feng Wu and Renjie Chen**

Supporting Information

Interface Engineering with Dynamics-mechanics Coupling for Highly Reactive and Reversible Aqueous Zinc-ion Batteries

Qianqian Meng, Xiaoyu Jin, Nuo Chen, Anbin Zhou, Huirong Wang, Ning Zhang, Zhihang Song, Yongxin Huang, Li Li, Feng Wu, Renjie Chen**

Dr. Q. Meng, X. Jin, N. Chen, A. Zhou, H. Wang, N. Zhang, Z. Song, Prof. Y. Huang, Prof. L. Li, Prof. F. Wu, Prof. R. Chen
Beijing Key Laboratory of Environmental Science and Engineering
School of Materials Science & Engineering
Beijing Institute of Technology
Beijing 100081, China.
E-mail: huangyx@bit.edu.cn, chenrj@bit.edu.cn

Prof. Y. Huang, Prof. L. Li, Prof. F. Wu, Prof. R. Chen
Institute of Advanced Technology
Beijing Institute of Technology
Jinan 250300, China.

Prof. L. Li, Prof. F. Wu, Prof. R. Chen
Collaborative Innovation Center of Electric Vehicles in Beijing
Beijing 100081, China

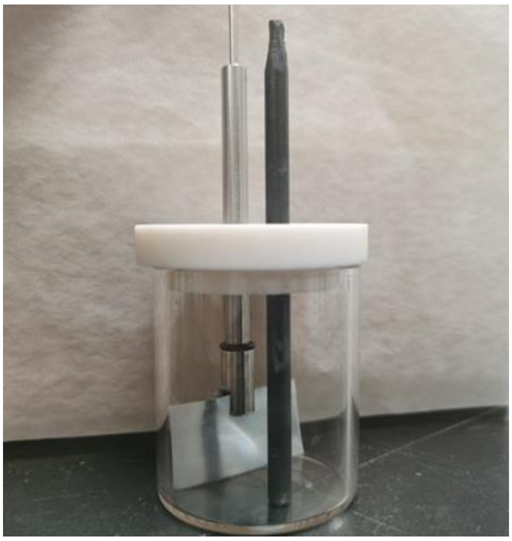

Figure S1. The optical photograph of electroplating device.

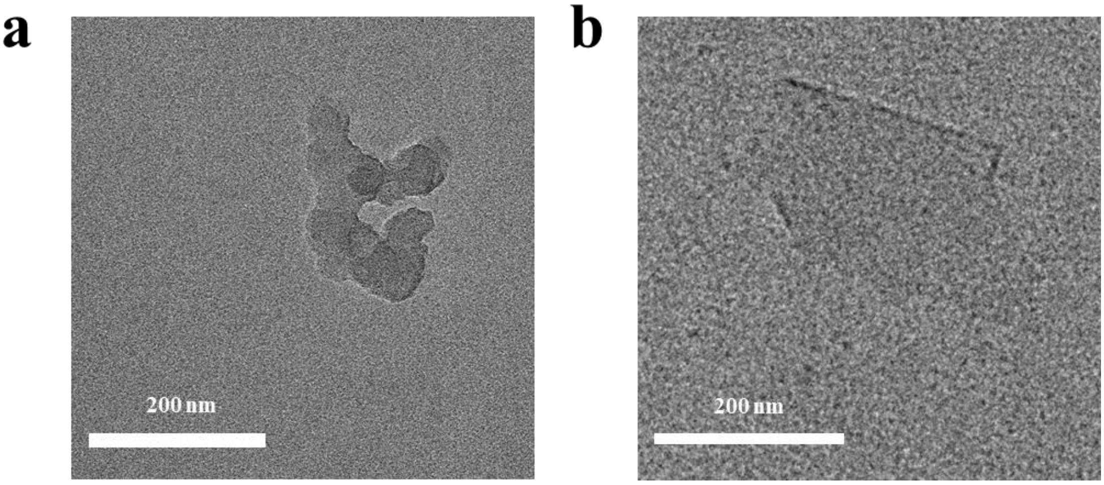

Figure S2. a,b) The TEM images of graphene and boronene nanosheets.

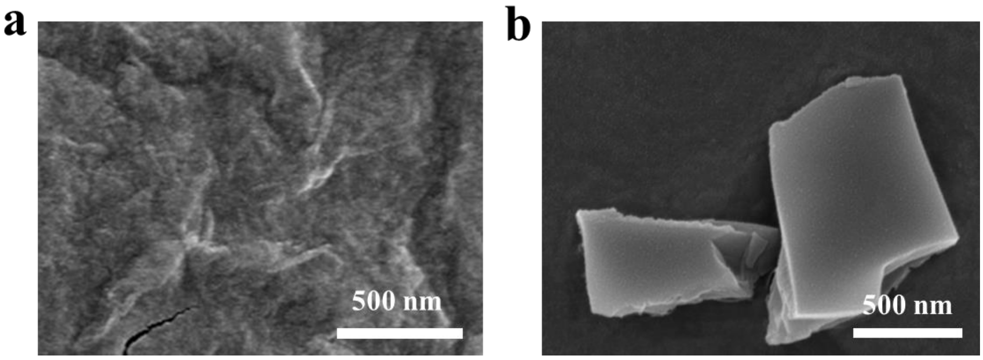

Figure S3. a,b) The SEM images of graphene and boronene nanosheets.

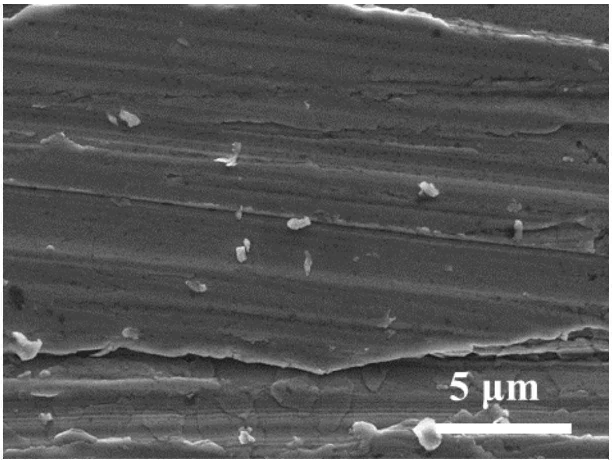

Figure S4. The SEM image of bare Zn.

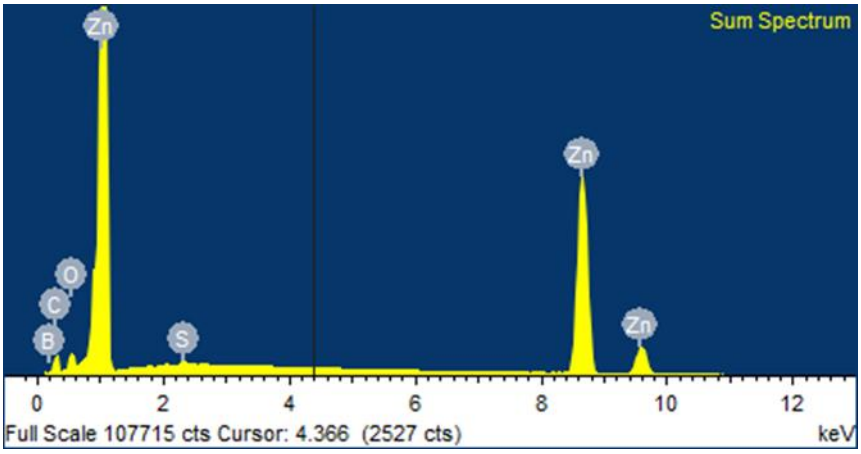

Figure S5. The element content mapping for the SEM image of G&B-S@Zn.

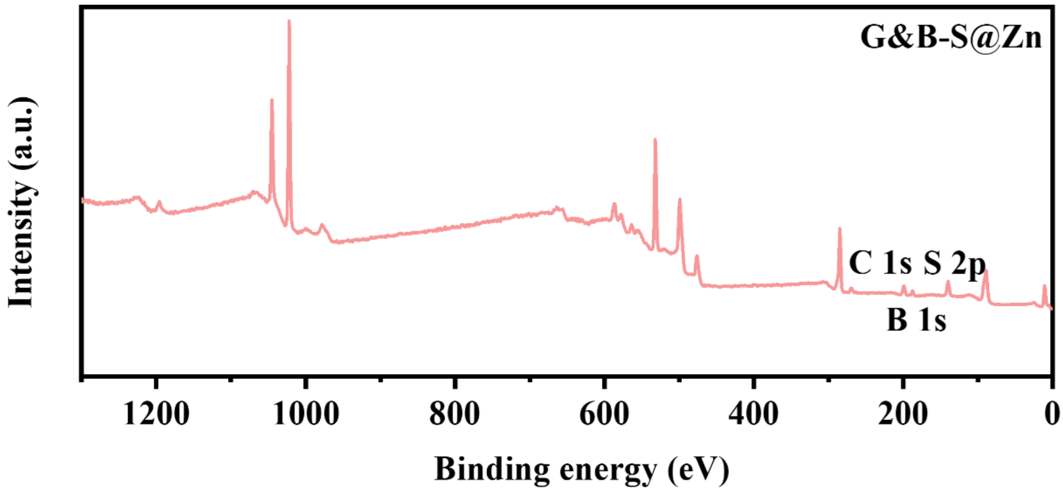

Figure S6. The XPS survey spectrum of G&B-S@Zn.

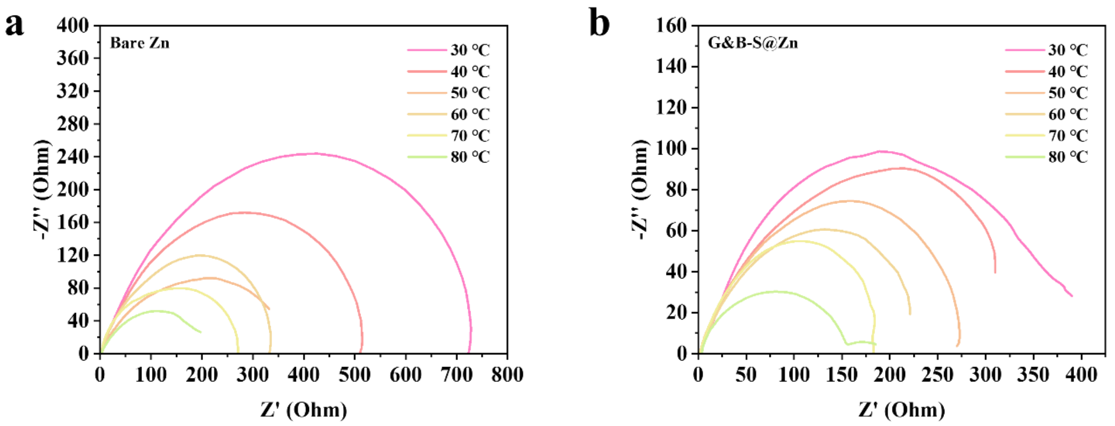

Figure S7. The Nyquist plots of a) bare Zn and b) G&B-S@Zn symmetric batteries in 30, 40, 50, 60, 70 and 80°C.

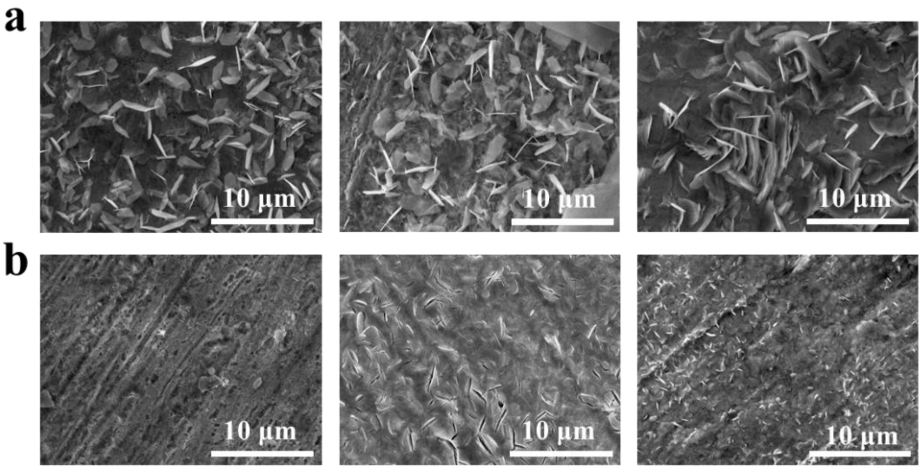

Figure S8. The SEM images of a) bare Zn and b) G&B-S@Zn immersed in 2 M $ZnSO_4$ aqueous solution for 3, 5 and 7 days.

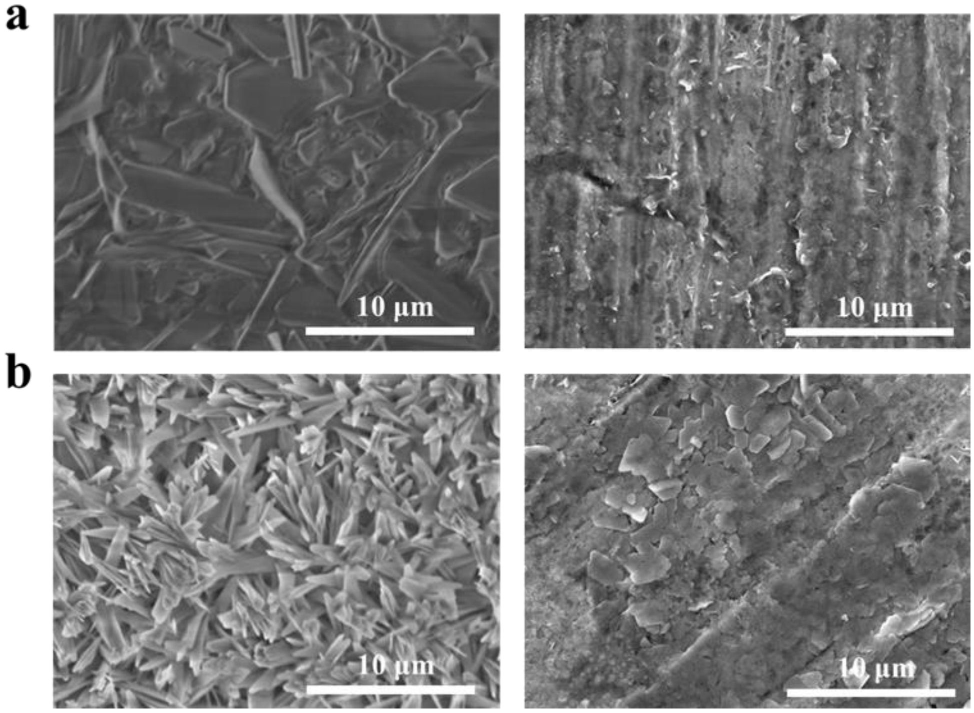

Figure S9. SEM images of bare Zn (left) and G&B-S@Zn (right) immersed in 1 M $ZnSO_4$ (a) and 1 M KOH (b) aqueous solution for 10 days.

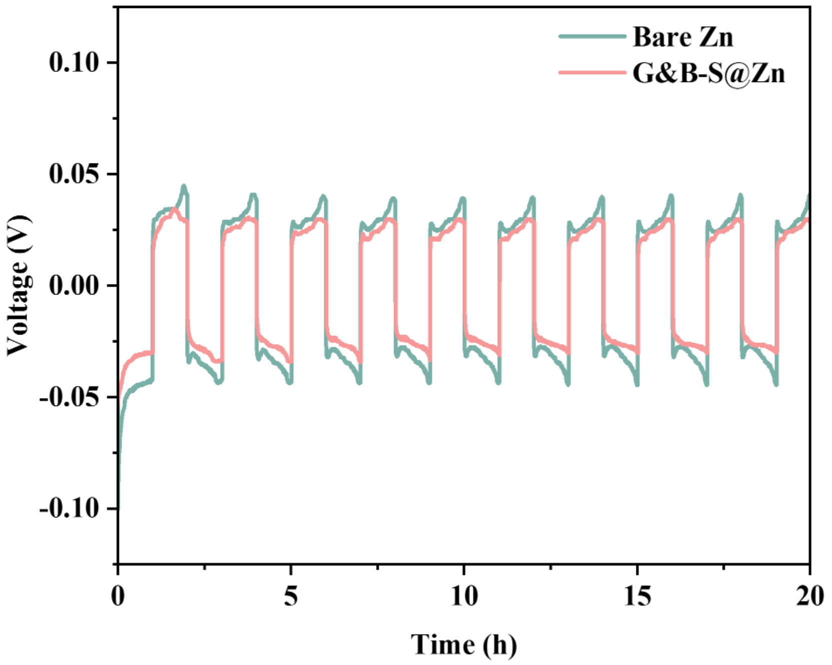

Figure S10. Electrochemical cycling curves of bare Zn and G&B-S@Zn corresponding to in-situ XRD.

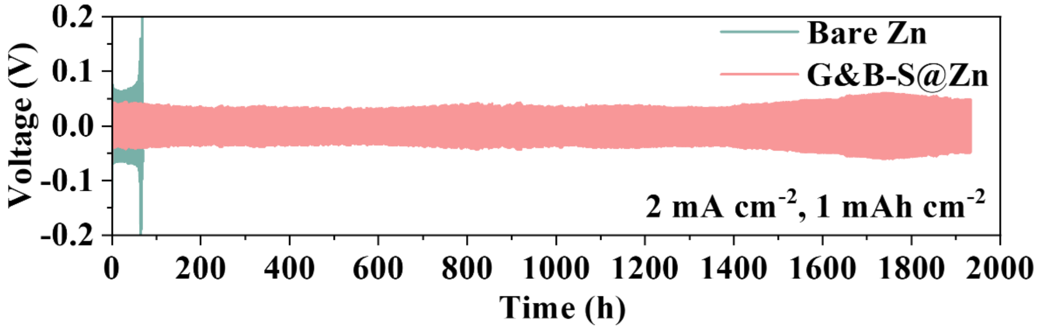

Figure S11. Galvanostatic cycling performance of bare Zn and G&B-S@Zn symmetric batteries at 2 mA cm^{-2} for 1 mAh cm^{-2}.

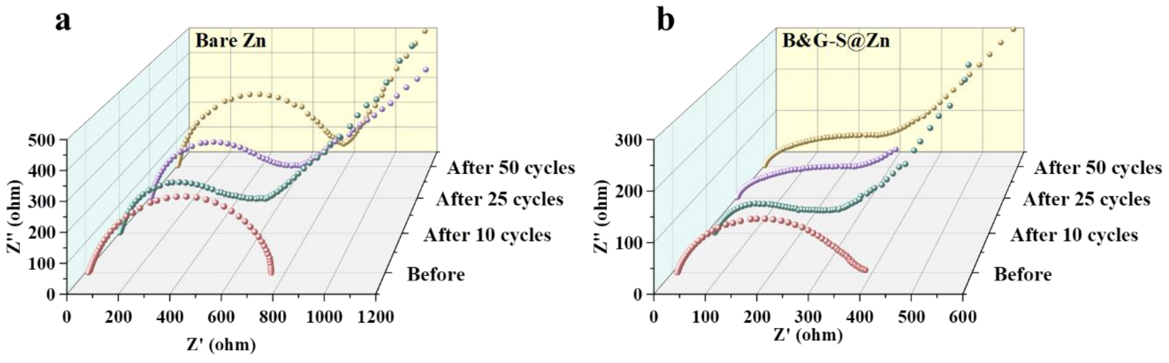

Figure S12. Nyquist plots of symmetric batteries at various cycles.

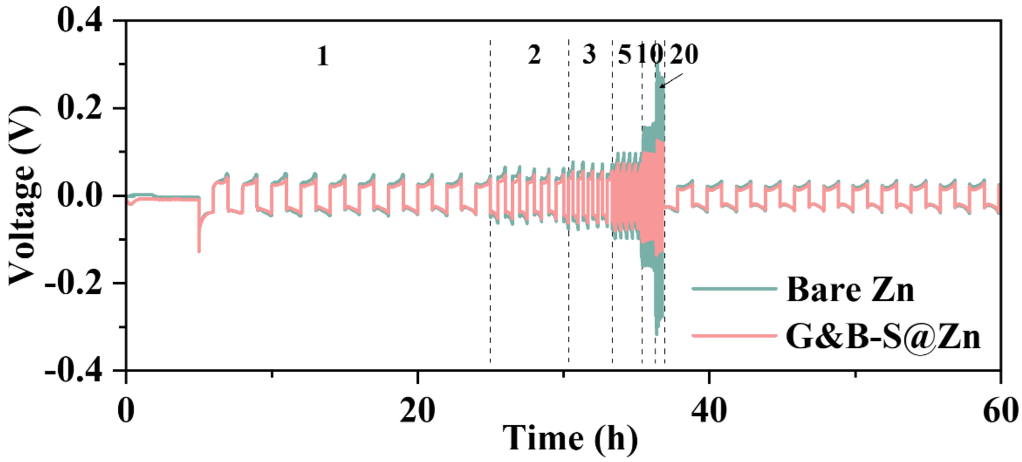

Figure S13. Rate performance of bare Zn and G&B-S@Zn at different current densities for a fixed capacity of 1 mAh cm^{-2}.

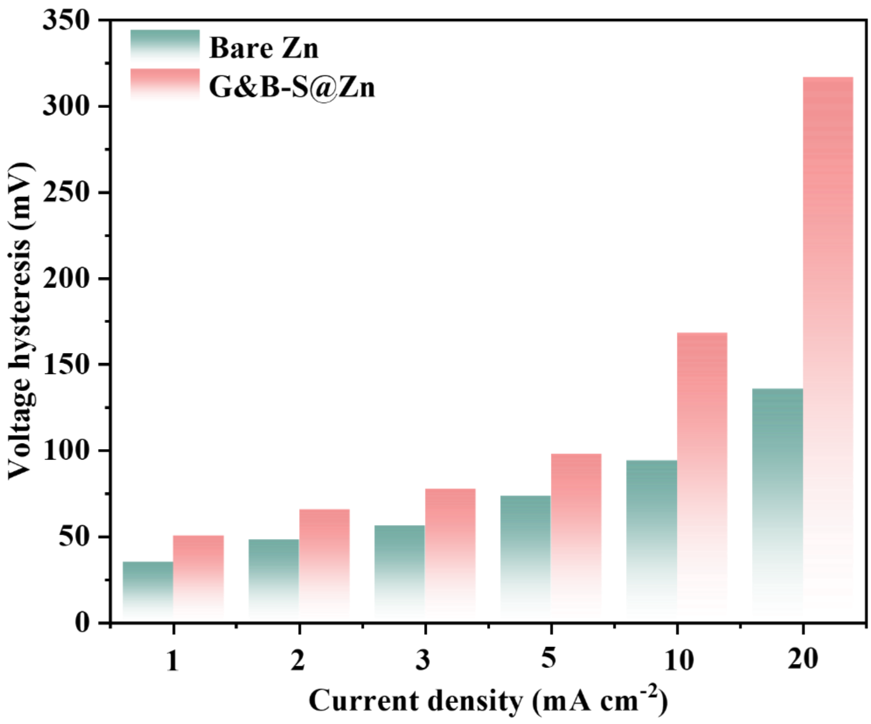

Figure S14. The corresponding voltage hysteresis for rate performance of bare Zn and G&B-S@Zn symmetric batteries.

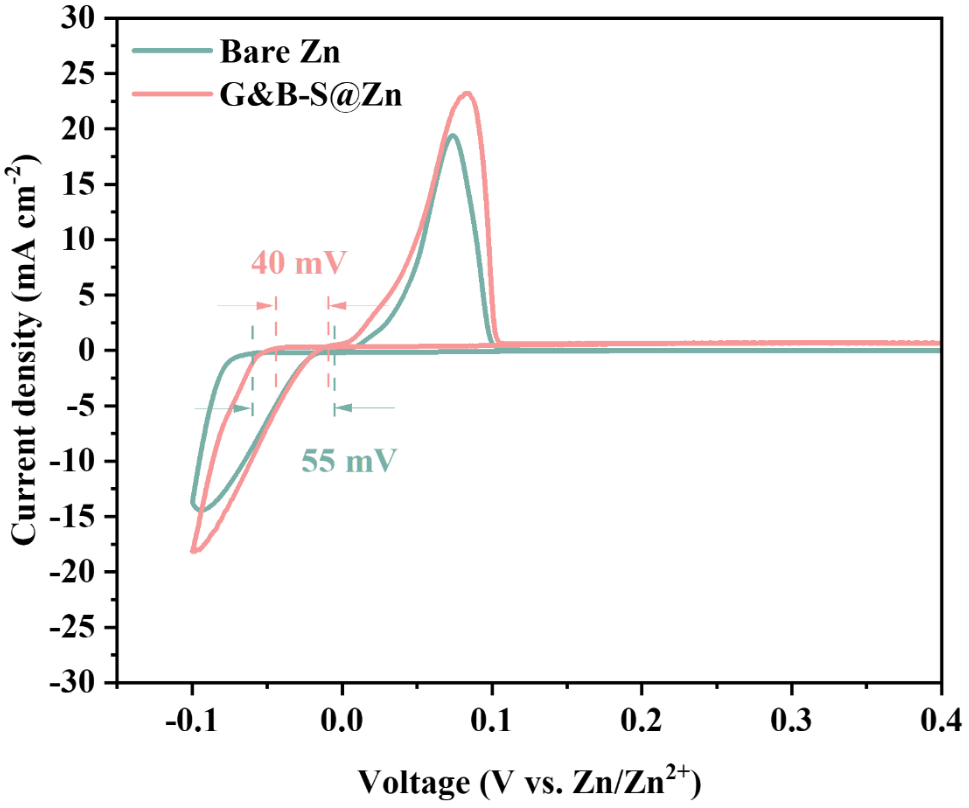

Figure S15. CV curves of bare Zn//Cu and G&B-S@Zn//Cu at a scan rate of 0.1 mV s^{-1}.

Table S1. Comparison of electrochemical performance between this work and graphene and layered-based interface

Interfacial Materials	Synthetic Method	Current Density (mA cm^{-2})	Area Capacity (mAh cm^{-2})	Cycle Life (h)	Average Voltage Hysteresis (mV)	Ref.
NGO@Zn	Langmuir–Blodgett Method	5	5	300	142	[1]
		1	1	1200	31	
S-BN@Zn	Spray Coating Method	5	2.5	1000	~50	[2]
		10	10	300	~75	
VG@Zn	Chemical Vapor Deposition	0.5	0.5	300	/	[3]
		1	1	250	/	
		5	5	70	/	

Zn-G	Coating Method	0.1	0.1	200	28	[4]
		0.2	0.2	300	~15	
Zn/rGO	Chemical Method	0.4	0.4	300	~15	[5]
		1	1	300	~15	
$Ti_3C_2Cl_2$−Zn	Scraping Method	2	1	840	40.7	[6]
		10	1	100	103	
MGA@Zn	Oriented Freeze-drying Method	10	1	1050	64	[7]
		20	1	27	/	
ZnGaIn//MXene	Blade Coating Method	1	1	600	25	[8]
		8	1	/	60	
ZGL@Zn	Doctor Blade Method	0.5	0.5	2180	/	[9]
GDY@Zn	Radio Frequency (RF) Heating Method	4	4	400	/	[10]
		2	2	~1800	/	
g-C_3N_4@Zn	3D printing Method	2	2	500	~70	[11]
		10	5	120	~200	
Zn-TiO_2	Blade Coating Method	0.18	0.045	200	~30	[12]
		2	0.5	200	~40	
		4.4	1.1	500	36.5	
Al_2O_3@Zn	Atomic layer Deposition Method	1	1	500	36.5	[13]
Zn@Zn-$Sb_3P_2O_{14}$	Coating Method	1	1	1300	~40	[14]
		10	10	450	~60	
UiO-67-2D@Zn	Drop-Casting Method	0.5	0.5	850	~25	[15]

		2	2	150	~25	
		5	5	60	~50	
		2	1	1900	~40	
G&B-S@Zn	Electroplating Method	5	1	1900	~30	This work
		5	2	1200	~20	
		20	1	600	112	

[1] J. Zhou, M. Xie, F. Wu, Y. Mei, Y. Hao, R. Huang, G. Wei, A. Liu, L. Li, R. Chen, Adv. Mater. **2021**, 33, 2101649.
[2] M. Qiu, H. Jia, C. Lan, H. Liu, S. Fu, Energy Stor. Mater. **2022**, 45, 1175.
[3] C. Li, Z. Sun, T. Yang, L. Yu, N. Wei, Z. Tian, J. Cai, J. Lv, Y. Shao, M. H Rümmeli, J. Sun, Z. Liu, Adv. Mater. **2020**, 32, 2003425.
[4] Z. Li, L. Wu, S. Dong, T. Xu, S. Li, Y. An, J. Jiang, X. Zhang, Adv. Funct. Mater. **2021**, 31, 2006495.
[5] A. Xia, X. Pu, Y. Tao, H. Liu, Y. Wang, Appl. Surf. Sci, **2019**, 481, 852.
[6] X. Li, M. Li, K. Luo, Y. Hou, P. Li, Q. Yang, Z. Huang, G. Liang, Z. Chen, S. Du, Q. Huang, and C. Zhi, ACS Nano, **2022**, 16, 813–822.
[7] J. Zhou, M. Xie, F. Wu, Y. Mei, Y. Hao, L. Li, R. Chen, Adv. Mater. **2022**, 34, 2106897.
[8] J. Gu, Y. Tao, H. Chen, Z. Cao, Y. Zhang, Z. Du, Y. Cui, S. Yang, Adv. Mater. **2022**, 12, 2200115.
[9] H. Gan, J. Wu, R. Li, B. Huang, H. Liu, Energy Stor. Mater. **2022**, 47, 602.
[10] C. Yin, M. Zhu, Y. Kong, Q. Wang, H. Zhou , L. Qi, L. Tong, J. Zhang, 2D Mater. **2021**, 8, 044003.
[11] P. Liu, Z. Zhang, R. Hao, Y. Huang, W. Liu, Y. Tan, P. Li, J. Yan, K. Liu, Chem. Eng. J. **2021**, 403, 126425.
[12] X. Zhou, P. Cao, A. Wei, A. Zou, H. Ye, W. Liu, J. Tang, J. Yang, ACS Appl. Mater. Interfaces, **2021**, 12, 8181.
[13] H. He, H. Tong, X, Song, X. Song, J. Liu, J. Mater. Chem. A, **2020**, 8, 7836.
[14] Y. Zhang, M. Zhu, G. Wang, F.-H. Du, F. Yu, K. Wu, M. Wu, S.-X. Dou, H.-K. Liu, C. Wu, Small Methods, **2021**, 5, 2100650.
[15] L. Lei, F. Chen, Y. Wu, J. Shen, X.-J. Wu, S. Wu, S. Yuan, Sci China Chem, **2022**, 6, 2205.

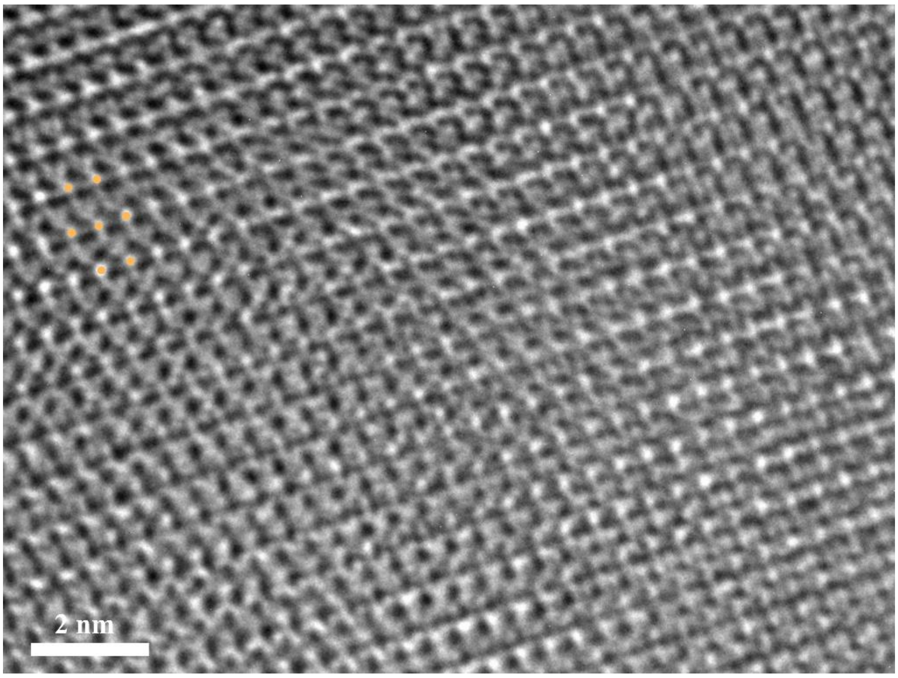

Figure S16. The high-resolution transmission electron microscopy (HRTEM) image of boronene.

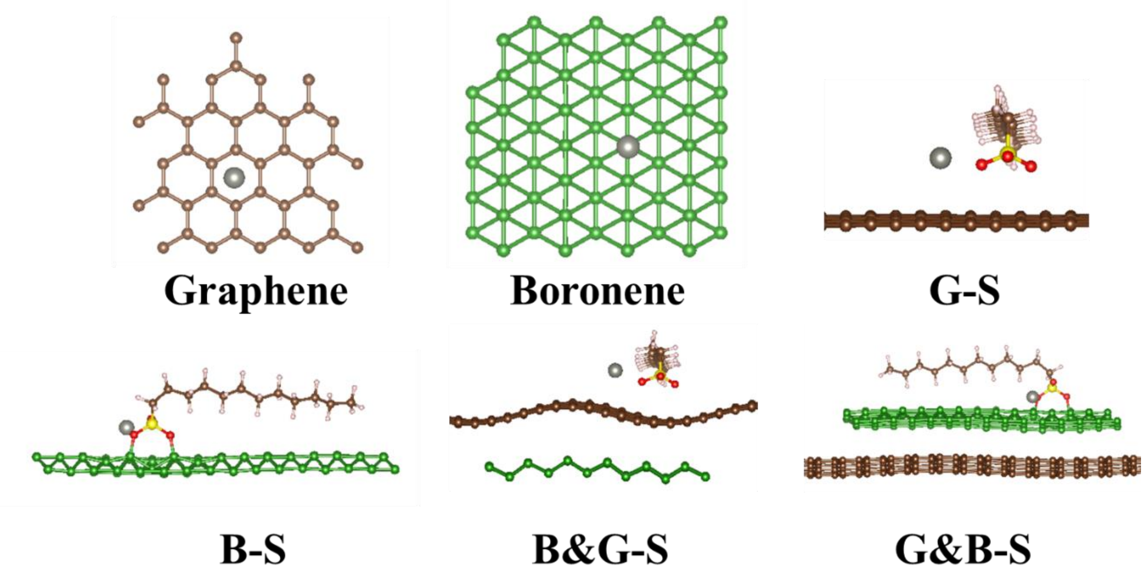

Figure S17. The adsorption configurations of Zn atom on graphene, boronene, G-S, B-S, B&G-S, and G&B-S structure from the top view.

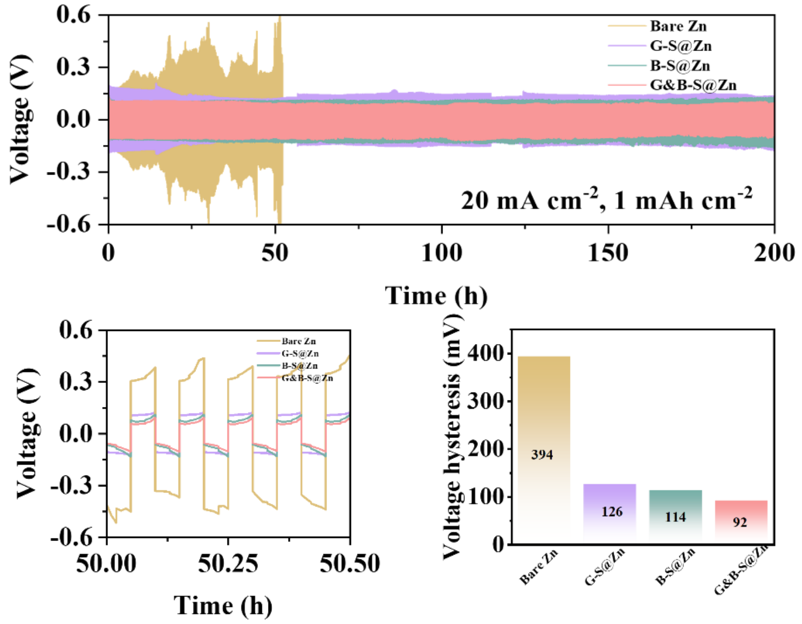

Figure S18. Galvanostatic cycling performance (a,b) and the the corresponding voltage hysteresis (c) of bare Zn, G-S@Zn, B-S@Zn, and G&B-S@Zn symmetric batteries at 20 mA cm^{-2} for 1 mAh cm^{-2}.

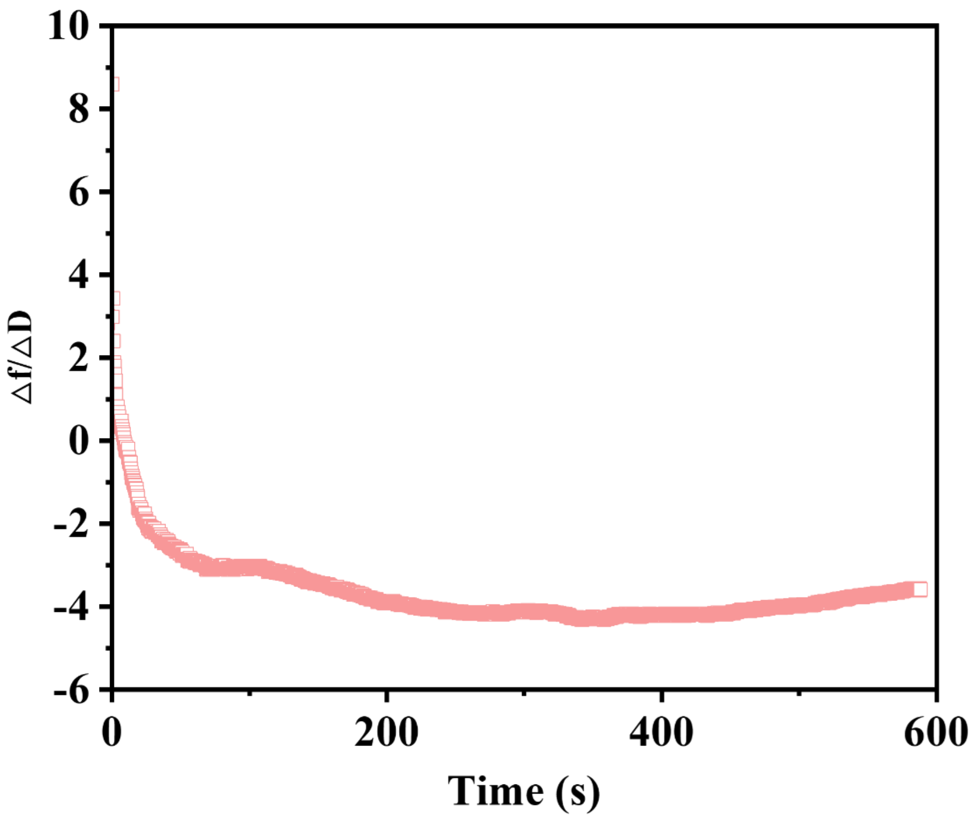

Figure S19. Δf/ΔD of the crystal-adsorbed G&B-S layer in QCM-D measurement.

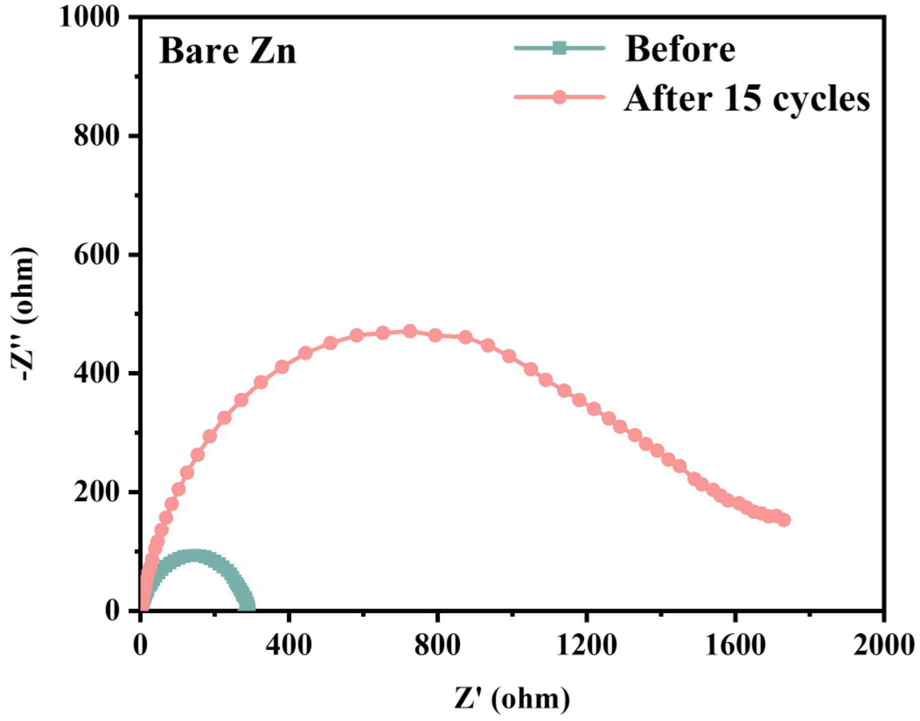

Figure S20. Nyquist plots of LMO// bare Zn cell.
